# Comparative metagenomics at Solfatara and Pisciarelli hydrothermal systems in Italy reveal that ecological differences across substrates are not ubiquitous

**DOI:** 10.3389/fmicb.2023.1066406

**Published:** 2023-02-01

**Authors:** Ifeoma R. Ugwuanyi, Marilyn L. Fogel, Roxane Bowden, Andrew Steele, Giuseppe De Natale, Claudia Troise, Renato Somma, Monica Piochi, Angela Mormone, Mihaela Glamoclija

**Affiliations:** ^1^Department of Earth and Environmental Sciences, Rutgers University, Newark, NJ, United States; ^2^EDGE Institute, University of California, Riverside, Riverside, CA, United States; ^3^Earth and Planets Laboratory, Carnegie Institution for Science, Washington, DC, United States; ^4^Istituto Nazionale di Geofisica e Vulcanologia, Osservatorio Vesuviano, Naples, Italy; ^5^Consiglio Nazionale delle Ricerche INO, Naples, Italy; ^6^Consiglio Nazionale delle Ricerche IRISS, Naples, Italy

**Keywords:** hydrothermal system, Solfatara, Pisciarelli, microbial diversity, metagenomics, MAGs

## Abstract

**Introduction:**

Continental hydrothermal systems (CHSs) are geochemically complex, and they support microbial communities that vary across substrates. However, our understanding of these variations across the complete range of substrates in CHS is limited because many previous studies have focused predominantly on aqueous settings.

**Methods:**

Here we used metagenomes in the context of their environmental geochemistry to investigate the ecology of different substrates (i.e., water, mud and fumarolic deposits) from Solfatara and Pisciarelli.

**Results and Discussion:**

Results indicate that both locations are lithologically similar with distinct fluid geochemistry. In particular, all substrates from Solfatara have similar chemistry whereas Pisciarelli substrates have varying chemistry; with water and mud from bubbling pools exhibiting high SO_4_^2−^ and NH_4_^+^ concentrations. Species alpha diversity was found to be different between locations but not across substrates, and pH was shown to be the most important driver of both diversity and microbial community composition. Based on cluster analysis, microbial community structure differed significantly between Pisciarelli substrates but not between Solfatara substrates. Pisciarelli mud pools, were dominated by (hyper)thermophilic archaea, and on average, bacteria dominated Pisciarelli fumarolic deposits and all investigated Solfatara environments. Carbon fixation and sulfur oxidation were the most important metabolic pathways fueled by volcanic outgassing at both locations. Together, results demonstrate that ecological differences across substrates are not a widespread phenomenon but specific to the system. Therefore, this study demonstrates the importance of analyzing different substrates of a CHS to understand the full range of microbial ecology to avoid biased ecological assessments.

## Introduction

Continental hydrothermal systems (CHSs) are usually formed within diverse igneous lithologies (i.e., mafic, andesitic, and felsic) and the hydrothermal fluids derived from deep subsurface sources may be mixed with marine, brine, or meteoritic water resulting in geochemically diverse environments. These diverse environments exhibit substantial differences in the availability and abundance of electron acceptors and donors (Fournier, 1989; Shock et al., 2010; Lowenstern et al., 2015; Lindsay et al., 2018; Amenabar and Boyd, 2019), which microorganisms exploit as sources of energy (Amenabar et al., 2017; Lindsay et al., 2019; Puopolo et al., 2020; Aulitto et al., 2021).

CHSs have been the subject of many studies as they are a surface manifestation of hydrothermal activities, which are easily accessible for diverse microbiological studies (Inskeep et al., 2013; Menzel et al., 2015; Colman et al., 2016; Mardanov et al., 2018; Oliverio et al., 2018; Power et al., 2018; Boyer et al., 2020; Podar et al., 2020; Crognale et al., 2022 as a few examples). These studies have revealed that hot springs support microbial communities that are exceptionally diverse and vary in their response to physical and geochemical parameters (Inskeep et al., 2013; Sharp et al., 2014; Lindsay et al., 2018; Power et al., 2018; Colman et al., 2019a,b; Podar et al., 2020). Some studies have found pH to be the primary physical parameter that influences the microbial community composition of hot springs in Yellowstone National Park (Boyd et al., 2010; Inskeep et al., 2013; Colman et al., 2016, 2019a,b), Tengchong, China (Hou et al., 2013; Xie et al., 2015), Iceland (Moreras-Marti et al., 2021), and volcanic provinces in New Zealand (Power et al., 2018). In contrast, other studies have identified temperature as the most important driver of microbial community composition in hot springs (Miller et al., 2009; Cole et al., 2013; Sharp et al., 2014; Podar et al., 2020). Further, at Yellowstone National Park, subsurface processes including phase separation and mixing with meteoritic fluids were shown to shape the ecology of hot spring communities through their influence on the availability of nutrients that support microbial metabolism (Lindsay et al., 2018; Colman et al., 2019a). In addition to temperature, pH, phase separation and mixing of fluids, Fullerton et al. (2021) found that microbial diversity analyzed from fluids and associated sediments reflects the subsurface geological structures that fluids traverse, which in turn influences carbon cycling within the subduction zone in Costa Rica.

While we have learned a great deal about hot springs, their geochemical complexity and the observed ecological differences between water and hot spring deposits (Cole et al., 2013; Colman et al., 2016) highlight the need for studies analyzing different substrates (e.g., fumarolic deposits) to understand the full range of microbial ecology in CHSs. Some studies have analyzed microbial communities from different available substrates (Glamoclija et al., 2004; Ellis et al., 2008; Benson et al., 2011; Sharp et al., 2014; Wall et al., 2015; Medrano-Santillana et al., 2017; Crognale et al., 2018; Marlow et al., 2020; Arif et al., 2021); however, to the best of our knowledge, no CHSs study has used as many different substrates from the same hydrothermal system and correlated their environmental settings and metagenomes to characterize microbial ecology. The lack of comparison among different substrates (fumarolic deposits, water and mud from mud pools) may derive from the fact that not all substrates are present at all CHSs. In this study, we use metagenomes to investigate the microbial ecology in different substrates (i.e., water, mud and fumarolic deposits) from two CHSs in the context of their environmental geochemistry. The objectives of this study were to: (1) assess the microbial diversity of different CHSs and a variety of available substrates (2) identify the metabolic potential of these microbial communities in connection to environmental geochemistry.

**Figure 1:**
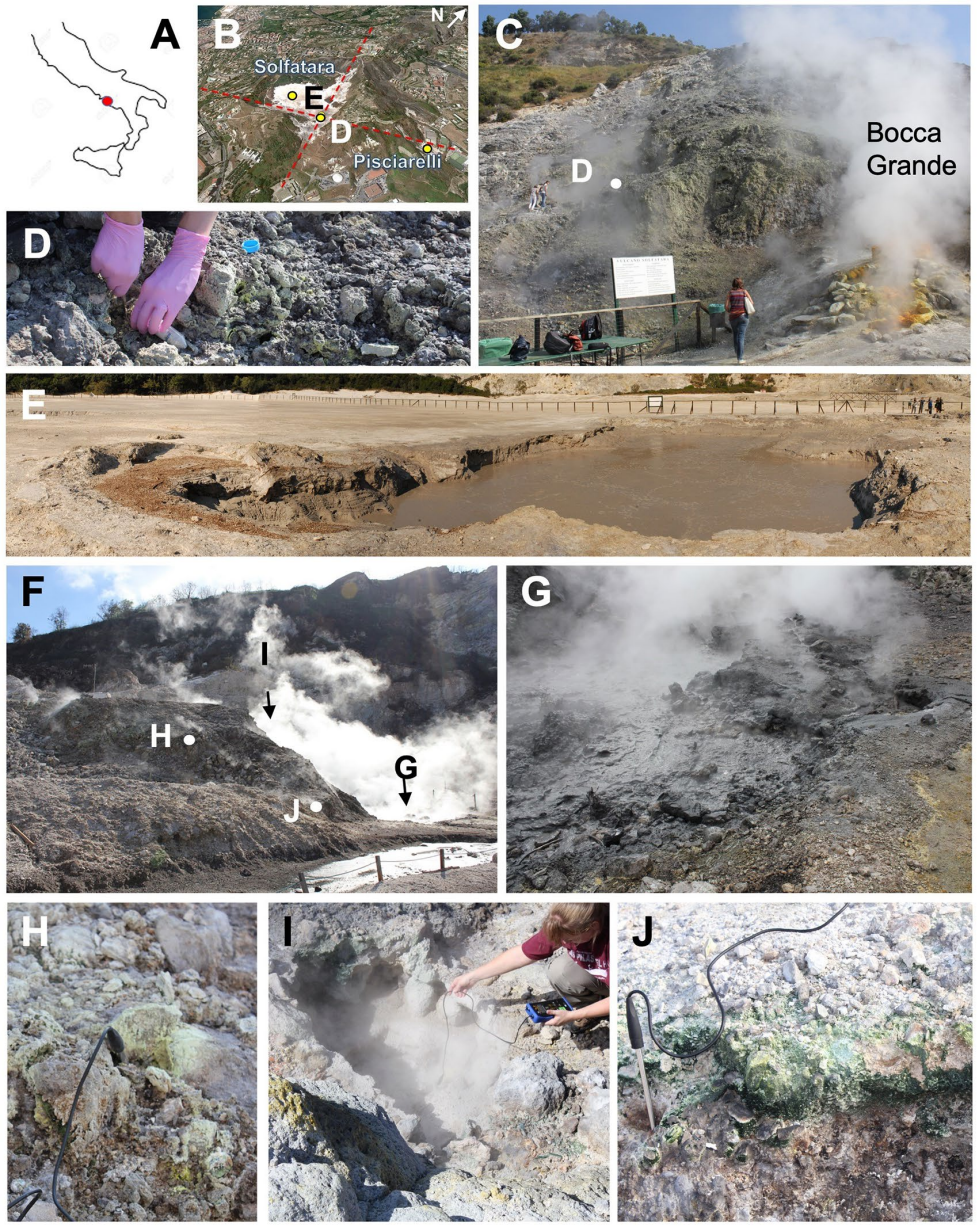
Sampling points at Solfatara-Pisciarelli hydrothermal systems. **(A)** Location map of Solfatara-Pisciarelli hydrothermal systems on contoured map of Italy (red dot). **(B)** Oblique view of Solfatara and Pisciarelli systems; red dashed lines represent faults; yellow dots are sampling points (E and D stand for Solfatara mud pool and fumaroles, respectively). **(C)** Sampling point of Solfatara fumarolic deposits (SF) next to the main fumarole Bocca Grande. **(D)** Close-up view of deposits to show heterogenicity of the material in regard to grain size and mineralogy (note different colors of deposits). **(E)** Solfatara mud pool (SMP) with bubbling water and warm muddy substrate near the pool. **(F)** Overview of the Pisciarelli location with marked sampling points. **(G)** Pisciarelli large pool (PLP) with bubbling hot mud (temp. 84.1°C). **(H)** Pisciarelli fumarolic deposits (PF) with sulfur crystals precipitate. **(I)** Pisciarelli small pool (PSP) sampling point. **(J)** Pisciarelli epilithic microbial layer (PLP-E) observed and sampled on the wall near the outflow channel of the large pool.

The study areas are Solfatara and Pisciarelli CHSs (Figure 1) located within Campi Flegrei Caldera (CFC); an 8 km nested caldera in Naples (Italy) formed by the Campania Ignimbrite (39 ka) and Neapolitan Yellow Tuff (15 ka) eruptions (De Natale et al., 2016; Rolandi et al., 2020a,b). Present-day activities at CFC are characterized by large-scale hydrothermal circulation, gaseous emissions, and intense ground deformation (Troise et al., 2019; Moretti et al., 2020; Chiodini et al., 2021). According to Caliro et al. (2007), fumaroles and mud pools at Solfatara and Pisciarelli are fed by fluids of mixed magmatic-meteoritic origin. The Solfatara crater is 0.6 km in diameter and maintains hydrothermal activity at fumaroles where gas emissions reach temperatures from 145°C up to 165°C, while mud pools have an average temperature of approximately 45°C (Glamoclija et al., 2004; Chiodini et al., 2011). Furthermore, the Solfatara environment is extremely acidic (mud pool: 1.9–2.1 pH and fumarolic deposits: 1.3–2.2 pH; Crognale et al., 2018). The second location, Pisciarelli, sits on the outside northeastern wall of the Solfatara crater. Pisciarelli holds an unstable fumarole field characterized by consistent deposit degassing, fluid emission from ephemeral vents, and boiling mud pools. This degassing activity is episodically accompanied by seismic swarms and macroscopic morphology changes such as the appearance of vigorously degassing vents, collapsing landslides, and bubbling mud (Fedele et al., 2021). Fumarolic deposits and mud pools at Pisciarelli are also very acidic (pH: 0.5–3) and temperatures of fumaroles ranges from 95°C to 110°C, while mud pools have temperatures between 84°C and 95°C (Ciniglia, 2005; Troiano et al., 2014; Poichi et al., 2019). In Solfatara-Pisciarelli CHSs, the major gases in fumarole vents are H_2_O and CO_2_ while the minor gases include H_2_S, N_2_, H_2_, CH_4_, He, Ar, and CO (Chiodini et al., 2010; Aiuppa et al., 2013). In both systems, fumaroles have a similar content of H_2_O, CO_2_, Ar, He, and N_2_, however, they differ in their concentration of H_2_S, H_2_, and CO (Chiodini et al., 2001). Solfatara and Pisciarelli also differ in their fluid geochemistry; fluid in mud pools of Pisciarelli have been reported to have higher NH_4_^+^ concentrations (508–1,026 mg L^−1^) compared to Solfatara (<1 mg L^−1^; Martini et al., 1991; Valentino and Stanzione, 2003; Glamoclija et al., 2004; Poichi et al., 2019) resulting in the precipitation of the minerals mascagnite [(NH_4_)_2_SO_4_] and tschermigite [(NH_4_)Al(SO_4_)_2_·12(H_2_O)] (Poichi et al., 2019). The geochemical diversity of substrates in Solfatara-Pisciarelli CHSs provides an excellent location for evaluating the composition, structure, and functional potential of thermophilic microorganisms in different hydrothermal substrates and locations.

## Materials and methods

To investigate the near-surface microbial community composition, structure and function at two CHSs, water, mud, and fumarolic deposits samples were collected from Solfatara and Pisciarelli (Figures 1A,B), in October 2012 (Supplementary Method). All samples were collected in triplicates, aseptically, using sterile Falcon tubes, Nalgene bottles, scoops, and gloves. After the collection, the samples were stored at −20°C until further processing; samples for long term storage were stored at −80°C. Temperature, pH, and redox potential (Eh) were measured *in situ* using a portable probe (Table 1). Gas readings were taken from the continuous Istituto Nazionale di Geofisica e Volcanologia (INGV) gas monitoring station at Solfatara and Pisciarelli (Table 1).

**Table 1:** Field measurements for water (W), mud (M), mud outlet (MO), epilith from dry mud wall (E), and fumarolic deposits (D) samples collected from Solfatara-Pisciarelli hydrothermal systems.

Location	Sample	T (°C)	pH	Eh (mV)	CO_2_ (%)	CH_4_ (ppm)	He (ppm)	H_2_ (ppm)	N_2_ (ppm)	Ar (ppm)	H_2_S (ppm)
Pisciarelli	PLP	84.1	2.5	−485							
	PLP-MO	78.9	2.5	−486							
	PLP-E	74	–	−501	98.43	259	9	870.28	7,686	110	4,496
	PSP	88.8	1.5	−628							
	PSP-M	94.6	2	−622							
	PF-D	93.3	1.5	−622							
Solfatara	SMP-W	42.5	1	330							
	SMP-M	68.8	1	330	24.1	13	2.35	490	469	0.39	1,250
	SF-D	88.7	1	198							

The gas readings were taken close to the fumarolic emission sites; at Solfatara, the gas monitoring station was near our sampling site for SF-S, and at Pisciarelli, near sampling site for PF-S2. Pisciarelli large pool (PLP), Pisciarelli small pool (PSP), Pisciarelli fumarole (PF), Solfatara mud pool (SMP), and Solfatara fumarole (SF).

### Geochemical characterization

About 1 g of solid sample (fumarolic deposits and mud) was added to 20 ml of MilliQ water and extracted as described in Lezcano et al. (2019). The concentration of water-soluble anions (F^−^, Cl^−^, NO_2_^−^, Br^−^, NO_3_^−^, PO_4_^3−^, and SO_4_^2−^) in extracts and water samples was measured in triplicate using an 881 compact IC pro ion chromatography system (Metrohm, Switzerland) with a Metrosep A Supp 5-250/4.0 column. The concentration of NH_4_^+^ in extracts and water samples was measured using the alkaline hypochlorite/phenol nitroprusside method, after adding sodium citrate to prevent the precipitation of calcium and magnesium salts (Solorzano, 1969). Before measuring concentrations of NH_4_^+^ in water samples, samples were diluted 10-fold. Ammonium sulfate (NH_4_)_2_SO_4_ solutions (0, 660, 1,320, 3,300, 6,600, and 13,200 ppm) were prepared and used as standard. The absorbance of each sample was measured in triplicate using an Evolution 60S UV-Vis Spectrophotometer at 640 nm wavelength.

X-ray fluorescence (XRF) was used to examine the chemical composition of mud and fumarolic deposits samples. Samples were analyzed in triplicate using a Horiba XGT-1000WR X-ray Fluorescence with an Rh tube X-ray source and elemental wt.% was determined using the XGT-1000WR software’s quantification (Supplementary Method).

### Stable isotopic analysis of carbon, nitrogen, and sulfur

Carbon, nitrogen, and sulfur stable isotope and elemental concentration analyses were performed at the Earth and Planets Laboratory, Carnegie Institution for Science. For organic carbon (TOC), and organic δ^13^C measurements, samples were weighed into silver boats and fumed with 12 N HCl for 12–14 h. δ^13^C and δ^15^N isotopes were measured using a Thermo Scientific Delta VPlus isotope ratio gas-source mass spectrometer connected to a Carlo Erba (NA 2500) elemental analyzer (EA/IRMS) *via* a Conflo III interface. δ^34^S was analyzed by the same gas-source mass spectrometer but connected to an Elementar Americas vario Micro CUBE elemental analyzer (EA-IRMS) *via* a Conflo III interface. Stable isotope values are reported in standard delta notation as ‰ variations relative to: Pee Dee Belemnite (PDB) for δ^13^C, atmospheric N_2_ gas (AIR) for δ^15^N, Vienna Canyon Diablo Troctolite (V-CDT) for δ^34^S, and with an analytical error of ±0.1‰. In-house δ^13^C and δ^15^N standards were also used and calibrated against international and certified standards as well. Additional δ^34^S standards include the International Atomic Energy Agency reference materials IAEA-S-1 (δ^34^S = −0.3‰), and IAEA S3 (δ^34^S = −32.3‰), as well as NBS-123 (δ^34^S = −17.09‰), NBS-127 (δ^34^S = −21.17‰), and USGS-42 (δ^34^S = −7.84‰). A subset of the samples was analyzed in 2021 at the EDGE Stable Isotope Laboratory at the University of California Riverside to confirm the very negative nitrogen isotope values in some of these samples. USGS25 (δ^15^N = −30.41‰) and USGS40 (δ^15^N = −4.52‰) were used as two of our calibrating standards and confirmed the values measured earlier at the Earth and Planets Laboratory.

### DNA extraction, metagenomic sequencing and sequence processing

About 0.25 g of mud and fumarolic deposits was used for DNA extraction. Before DNA extraction, fumarolic deposits were powdered using a sterilized agate mortar and pestle. Water samples were filtered in the laboratory using a 0.2 μm VWR black polycarbonate filter. Filters were cut into small pieces with a sterile scalpel and used for DNA extraction. DNA was extracted using DNeasy PowerSoil kit (Qiagen Inc., Valencia, CA, United States) with modifications to the manufacturer’s instructions (Supplementary Method) and were stored at −80°C until further processing. Sequencing libraries were prepared using the Accel-NGS 2S Plus DNA Library Kit (Swift Biosciences Inc., Ann Arbor, MI, United States) according to the manufacturer’s protocols. The prepared library was sequenced on the Illumina Novaseq platform at the Oklahoma Medical Research Foundation. Metagenome sequence quality was assessed using FastQC v0.11.5^1^ and reads with a mean quality score less than 25 were discarded. Sequences were quality trimmed and Illumina sequencing adapters removed using Trimmomatic v0.36 (Bolger et al., 2014) with the following parameters: SLIDINGWINDOW:4:15, LEADING:3, TRAILING:3, and MINLEN:36. After quality control, taxonomic annotation of metagenomes was performed using Kaiju v1.8.2 (Menzel et al., 2016) against the NCBI nr + euk database (accessed on February 16, 2022) with the following parameters (run mode: greedy, minimum match length: 11, minimum match score: 75, allowed mismatches: 5). To compare the effects of taxonomic profiling using metagenome vs. 16S rRNA gene sequences, the trimmed metagenome sequences were uploaded to the MG-RAST online server (Meyer et al., 2008) and then passed through the MG-RAST QC pipeline. Following the quality control on MG-RAST, 16S rRNA gene sequences present in the metagenome (metagenome-derived 16S rRNA gene sequences) were identified and taxonomy assigned. Taxonomic classification of the metagenome-derived 16S rRNA gene sequences was done using the Greengenes rRNA database (DeSantis et al., 2006) hosted in MG-RAST with a minimum cut-off identity of 60% and e-value of 5. Since contamination leads to an overestimation of diversity, especially in low biomass samples like that of Solfatara and Pisciarelli (Karstens et al., 2019), after assigning taxonomy, we removed molecular biology kit and laboratory contaminants (which hereinafter are referred to as “kitome”), low abundance taxa (<0.01%) and sequences that were unclassified at the domain level from the taxonomy table before performing downstream analyses (Supplementary Method).

### Co-assembly, metagenomic binning, and MAG quality assessment

Bins were generated from 10 Solfatara and 17 Pisciarelli metagenomes. To maximize genome recovery, we co-assembled several samples based on their taxonomic composition. All 10 Solfatara metagenomes were co-assembled and all 17 Pisciarelli metagenomes were co-assembled using MEGAHIT v1.2.9 (Li et al., 2015). Contigs longer than 1,000 bp were then binned using the metaWRAP binning module (Uritskiy et al., 2018) which incorporates three binning methods: CONCOCT v1.1.0 (Alneberg et al., 2014), MaxBin2 v2.2.6 (Wu et al., 2016), and metaBAT2 v2.12.1 (Kang et al., 2019). The metaWRAP refinement module (Uritskiy et al., 2018) was used to merge results from the three binning methods using the -c 50 and -x 10 options to obtain bins with over 50% completeness and less than 10% contamination according to the CheckM tool v1.0.12 (Parks et al., 2015). Bins with >50% completeness and <10% contamination were then reassembled with SPAdes v3.13.0 (Bankevich et al., 2012) to improve the assembly quality. The contamination and completeness of resulting Metagenome Assembled Genomes (MAGs) were reassessed with CheckM (Parks et al., 2015).

### Taxonomic and functional annotation of MAGs

Taxonomy was assigned to MAGs using GTDB-TK v1.7.0 (Chaumeil et al., 2020). To assess whether MAGs belong to the same species (species ANI ≥ 95%), average nucleotide identity (ANI) was calculated for each possible pair of MAGs using FastANI v1.33 (Jain et al., 2018). MAGs with taxonomic assignment similar to previously identified kitome (e.g., *Corynebacterium* and *Staphylococcus*; Sheik et al., 2018; Weyrich et al., 2019) were not included in downstream analyses. Functional annotation of MAGs, including gene prediction was done using Prokka v1.14.5 (Seemann, 2014). Predicted genes were compared against the Kyoto Encyclopedia of Genes and Genomes (KEGG) database using BlastKOALA server (Kanehisa et al., 2016) to obtain KEGG Orthology (KO) annotations.

### Abundance of MAGs in metagenomes

To assess the abundance of recovered MAGs, quality trimmed sequences from each metagenome were mapped against each MAG using BBMap v38.96. Sequence counts were normalized as the number of sequences recruited per kilobase of MAG and gigabase of metagenome (RPKG). The normalized sequence counts allowed for direct comparison of genome abundance between metagenome of different depths.

### Statistical analyses

All statistical analyses were performed in R v4.0.2 (R Core Team, 2020). Principal Component Analysis (PCA) was performed with environmental parameters using the “*prcomp*” function in the stat package with scaling enabled. PCA results were graphed using the “*ggbiplot*” function from the ggbiplot v0.55 package. Before using the geochemical data for statistical analyses, variables below detection limit in all samples were excluded and the dataset transformed using z-score. After removing kitome and all potential contaminants, we calculated alpha diversity using the species abundance table obtained from metagenome-derived 16S rRNA gene (Greengenes) and metagenome (NCBI nr + euk) taxonomic assignment. Alpha diversity (Shannon, observed richness and Pielou’s evenness) was calculated using the “*estimate_richness*” function from the phyloseq v1.32.0 package in R. Nonpareil v3.0 (Rodriguez-R et al., 2018) was then used to estimate coverage and also calculate diversity with kmer kernel and default parameters. Kruskal-Wallis test was used to investigate whether alpha diversity varied significantly between locations and substrates. The “*lm()*” function in R was used to perform linear regression to evaluate potential effects of environmental variables on alpha diversity. Bray–Curtis dissimilarity based on genus and species abundance was used to determine differences in microbial community composition. The Bray-Curtis distance calculated was visualized using a Non-metric Multidimensional Scaling (NMDS) plot. Analysis of Similarity (ANOSIM) was performed on the Bray–Curtis dissimilarity matrix using the vegan v2.5-6 package to evaluate the significance of microbial compositional differences between the two locations and different types of substrates. Similarity Percentage (SIMPER) analysis was performed using the vegan v2.5-6 package to identify genera that contributed to the dissimilarity between two locations and different types of substrates. Mantel test using Spearman’s correlation coefficient with 999 permutations was performed to evaluate the significance of correlation between community composition (Bray–Curtis dissimilarity) and environmental parameters (Euclidean distance).

## Results and discussion

### Geochemical context of Solfatara-Pisciarelli hydrothermal systems

At Solfatara-Pisciarelli CHSs (Figure 1), the crater structure that forms the rock substrate (alkaline potassic tephra and lava ranging from trachybasalt to phonotrachyte) for both locations was produced and shaped by the same volcanic events (Piochi et al., 2015). Solid substrates from both locations have similar bulk elemental composition [Supplementary Figure 1; Supplementary Table 1 and Piochi et al. (2014), Piochi et al. (2015)]. In addition, the same magma chamber provides a heat source for the fumaroles and mud pools (Chiodini et al., 2001; Valentino and Stanzione, 2003) of both systems. However, tectonic features diverge the fluids that feed Pisciarelli mud pools to pass through old marine sediments with strong organic imprints where the fluids become enriched in NH_4_^+^ (Poichi et al., 2019).

**Figure 2:**
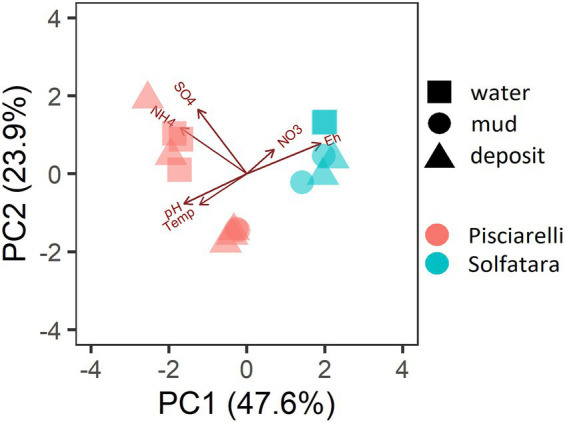
Biplot based on Principal Component Analysis (PCA) using temperature, pH, Eh, water-soluble anions and cations. Variables that had values below detection limit in most samples (F^−^, Cl^−^, and PO_4_^3−^) were excluded from PCA.

PCA based on temperature, pH, Eh, and water-soluble nutrients showed further distinction between Solfatara and Pisciarelli (Figure 2; Supplementary Table 2). Pisciarelli an extremely acidic (pH: 1.5–2.5) and reducing (Eh: −628 to −485) environment has higher temperatures (74°C to 95°C) and gas concentrations including H_2_S and CH_4_ (Table 1) compared to Solfatara (pH: ~1; temperature: 42°C to 88.7°C; Eh: 198 and 330 mV), making Pisciarelli a more active and extreme hydrothermal environment than Solfatara. These higher temperatures, which inhibit the solubility of oxygen (Boyer et al., 2020), together with higher concentrations of reducing gases may contribute to the more reducing conditions we observed at Pisciarelli. The H_2_S gas released in CHSs may be oxidized abiotically by oxygenated meteoritic waters (Nordstrom et al., 2005, 2009) or biotically by chemosynthetic microorganisms leading to the higher concentration of SO_4_^2−^ we observed in mud pools (Pisciarelli: 3,326–6,208 ppm; Solfatara: 206–2,888 ppm) compared to fumarolic deposits (Pisciarelli: 118–439 ppm; Solfatara: 567–610 ppm). The concentrations of SO_4_^2−^ in Pisciarelli mud pools were two times that of Solfatara mud pool possibly due to the higher concentration of H_2_S gas measured at Pisciarelli. The concentration of NH_4_^+^ was also higher in mud pools, particularly in Pisciarelli mud pools (Pisciarelli: 1,130–1,998 ppm; Solfatara: 1.1–36 ppm) than in fumarolic deposits (Pisciarelli: 0.8 to 38 ppm; Solfatara: 0.3 ppm) which corresponds with other studies that report higher concentrations of NH_4_^+^ in Pisciarelli mud pools (Martini et al., 1991; Valentino and Stanzione, 2003; Glamoclija et al., 2004; Poichi et al., 2019). Temperature and geochemical measurements from Pisciarelli large mud pool revealed an environmental gradient; values were lower at the discharge channel (temperature: 79°C; SO_4_^2−^: 134.4–193.6 ppm; NH_4_^+^: 46.5–67.8 ppm) compared to the main bubbling pool (temperature: 84°C; SO_4_^2−^: 3326–6,208 ppm; NH_4_^+^: 1,130–1,998 ppm). The low concentrations of SO_4_^2−^ and NH_4_^+^ along the discharge channel may result from the removal of H_2_S and NH_4_^+^ possibly due to oxidation and volatilization as fluid flows into the channel.

### Discrepancies between metagenome-derived 16S rRNA gene and metagenomic profiles

**Figure 3:**
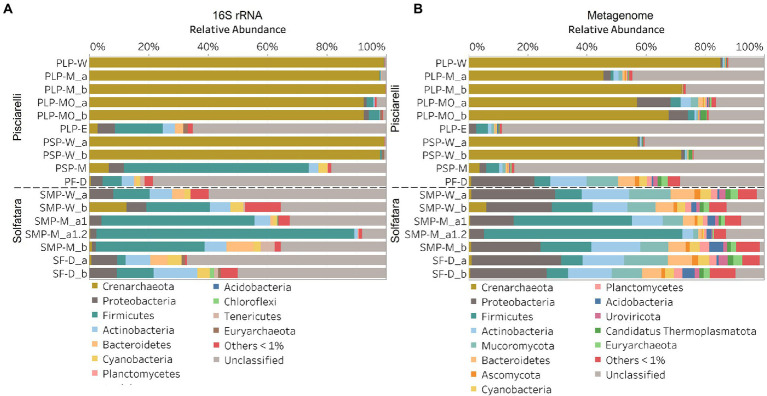
Relative abundance of phyla based on **(A)** metagenome-derived 16S rRNA gene and **(B)** metagenomic profiling. Relative abundance was calculated after removing sequences that were unclassified at the domain level, kitome, singleton, species present in only one sample and low abundance species. Phyla with average abundance of <1% were grouped into Others <1%. W represents water, M for mud, MO for mud outlet, E for epilithic microbial layer from dry mud, and D for fumarolic deposits samples. Samples labeled a and b are replicates sampled from the same spot. Sample SMP-M_a1 and SMP-M_a1.2 are sequencing replicates.

**Figure 4:**
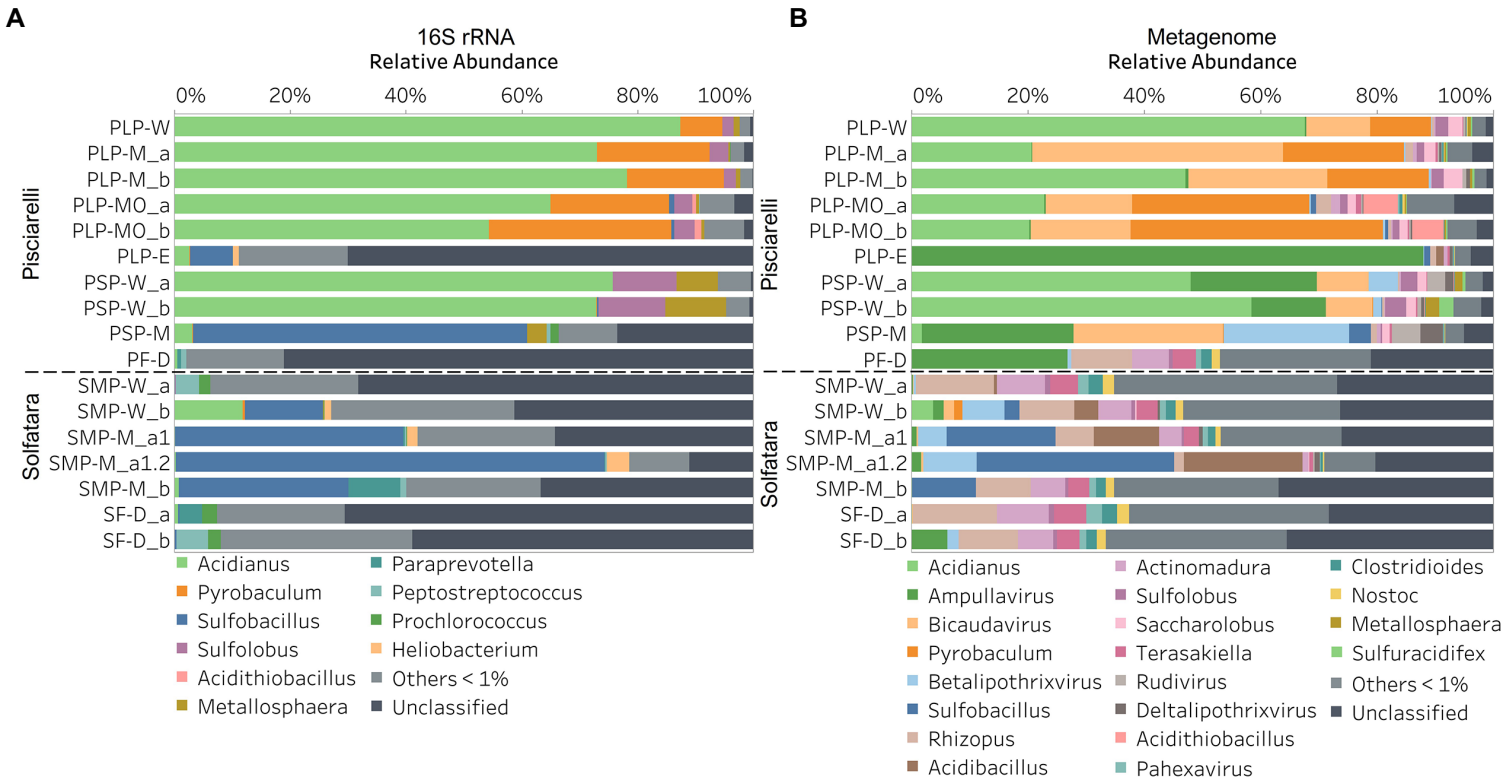
Relative abundance of genera based on **(A)** metagenome-derived 16S rRNA gene and **(B)** metagenomic profiling. Relative abundance was calculated after removing sequences that were unclassified at the domain level, kitome, singleton, species present in only one sample and low abundance species. Genera with average abundance of <1% were grouped into Others <1%. W represents water, M for mud, MO for mud outlet, E for epilithic microbial layer from dry mud, and D for fumarolic deposits samples. Samples labeled a and b are replicates sampled from the same spot. Sample SMP-M_a1 and SMP-M_a1.2 are sequencing replicates.

In this study, a total of 7 Solfatara and 10 Pisciarelli samples were analyzed. Solfatara samples had between 54,932,932 to 210,855,916 (average = 126,261,781) quality filtered metagenome sequences whereas Pisciarelli had between 119,199,732 to 230,316,988 (average = 179,177,105) quality filtered metagenome sequences. We compared taxonomic profiles generated from metagenome against metagenome-derived 16S rRNA gene sequences and observed that metagenome sequences identified more taxa than metagenome-derived 16S rRNA gene sequences, which corresponds to other studies that detected an increased number of taxa with whole genome shotgun sequencing compared to the 16S amplicon method (Ranjan et al., 2016; Brumfield et al., 2020). For example, Ranjan et al. (2016) reported that with the same number of sequences, whole genome shotgun sequencing identified twice as many species as the 16S method. We observed that metagenome sequences identified 56 phyla while metagenome-derived 16S rRNA gene sequences identified 26 phyla. Only 3 out of the 26 phyla detected by metagenome-derived 16S rRNA gene profiling were not detected by metagenomic profiling. Metagenomic profiling, on the other hand, identified 35 phyla not identified by metagenome-derived 16S rRNA gene profiling with 12 of them being viral and eukaryotic phyla. Overall, the dominant bacterial and archaeal phyla detected were similar irrespective of profiling method. Predominant bacterial and archaeal phyla detected by both profiling methods include *Crenarchaeota*, *Proteobacteria*, *Firmicutes*, and *Actinobacteria* (Figures 3A,B). At the genus level, metagenomic profiling also identified more genera than metagenome-derived 16S rRNA gene profiling (Figures 4A,B). Of the 245 genera identified across all samples by metagenomic profiling methods only 48 genera (20%) were identified with both profiling methods. Metagenomic profiling identified 197 genera not identified by metagenome-derived 16S rRNA gene profiling including *Acidilobus*, *Acidibacillus*, *Thermogymnomonas*, *Ampullavirus*, and *Bicaudavirus* while genera including *Acetobacterium* and *Caldococcus* were unique to metagenome-derived 16S rRNA gene profiling. We observed that both methods identified dominant genera including *Acidianus*, *Pyrobaculum*, and *Sulfobacillus*.

**Figure 5:**
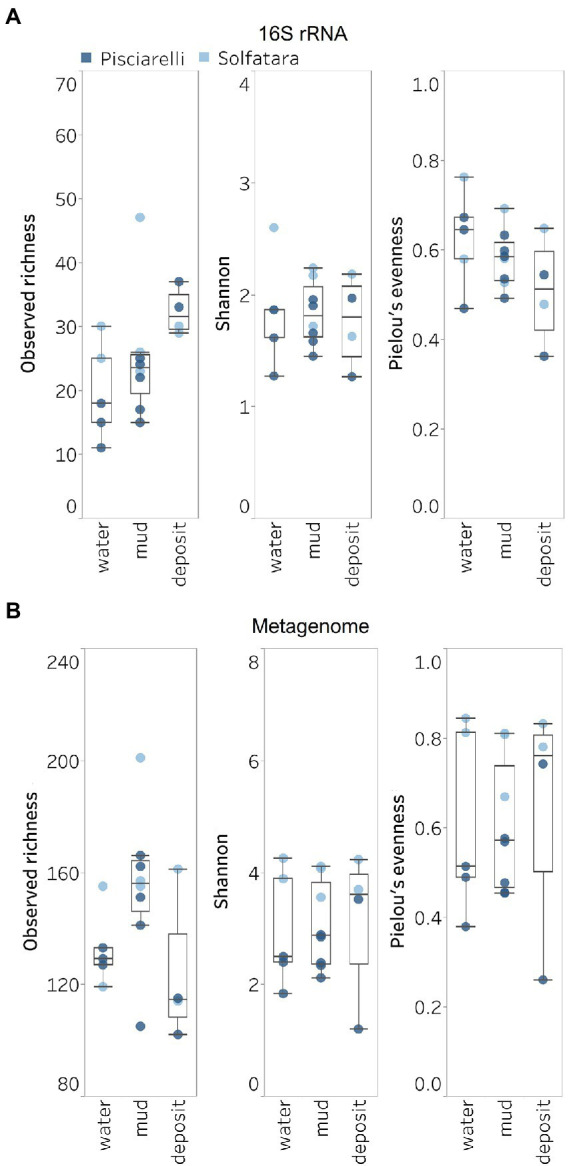
Alpha diversity across locations and substrate types calculated using species abundance from **(A)** metagenome-derived 16S rRNA gene and **(B)** metagenomic profiling.

Next, we analyzed the effect of both taxonomic profiling methods on diversity (Shannon diversity, observed richness, Pielou’s evenness). Before calculating the diversity, we identified and removed kitome (Supplementary Figure 3; Supplementary Tables 4, 5), low abundance taxa and sequences that were unclassified at the domain level from the taxonomy table to avoid an overestimation of diversity and a misrepresentation of community composition (Karstens et al., 2019). Our results showed that Shannon diversity and observed richness were consistently higher when the taxonomy table from metagenomic profiling was used as input compared to when we used the taxonomy table from metagenome-derived 16S rRNA gene profiling (Figures 5A,B). Overall, our results reflect that the 16S amplicon approach, which has been the most employed approach for studying CHSs’ microbiome, identifies a significantly lower number of bacterial species and completely excludes viruses and fungi which is similar to reports from other studies (Ranjan et al., 2016; Brumfield et al., 2020). Although metagenome sequencing permits the identification of more taxa, the choice between 16S rRNA gene and metagenome sequencing ultimately depends on the ecological questions and objectives of any given study. Since metagenome sequences encompass all members of the microbiome and provide high-resolution diversity analysis, which agrees with our objective to understand the full range of microbial ecology in available substrates from Solfatara-Pisciarelli CHSs, the further analyses and results hereinafter are based on the taxonomy table generated from metagenomic profiling.

### Microbial diversity of Solfatara-Pisciarelli hydrothermal systems

Shannon diversity in Solfatara-Pisciarelli CHSs ranged from 1.20 to 4.26 (Figure 5B; Supplementary Table 8). We observed significant variations in Shannon diversity (Kruskal–Wallis: *p* < 0.05) between Solfatara and Pisciarelli which were supported by Nonpareil diversity values (Supplementary Figure 4; Supplementary Table 8); on average, Shannon diversity was lower at Pisciarelli (Shannon: 1.20–3.52) than at Solfatara (Shannon: 3.55–4.26). Additionally, microbial communities were more uneven (Kruskal–Wallis: *p* < 0.05) at Pisciarelli (Pielou’s evenness: 0.26–0.74) than at Solfatara (Pielou’s evenness: 0.67–0.84). However, there was no significant difference in observed richness between these locations (Solfatara: 114–201 species; Pisciarelli: 102–166 species; Kruskal–Wallis: *p* = 0.24). The decreased Shannon diversity at Pisciarelli may have resulted from the combined effects of reducing conditions, extremely low pH, and high temperature. The addition of highly reducing conditions at Pisciarelli to an already extreme environment (high temperature and low pH) may cause further selection against certain microbial groups by hindering their growth and metabolism, thus leading to the lower Shannon diversity observed (Kimbrough et al., 2006; Seo and DeLaune, 2010; Husson, 2013; Zhang et al., 2015). Alpha diversity in substrates showed some variation (e.g., on average, mud and fumarolic deposits had higher Shannon diversity than water, and richness showed less variability in water and mud); however, these differences were not statistically significant (Kruskal–Wallis, Shannon: *p* = 0.97; Pielou’s evenness: *p* = 0.83; observed genus richness: *p* = 0.09) even when substrates were analyzed separately for each location which implies that species alpha diversity does not differ across substrates in the Solfatara-Pisciarelli CHSs. This result contrast with other studies comparing microbial diversity in water and solid substrates from hot springs, which showed differences in richness (Colman et al., 2016) and evenness (Cole et al., 2013) across substrates.

A comparison of diversity and physical parameters showed pH (Shannon: *R*^2^ = 0.73; Nonpareil: *R*^2^ = 0.43, *p* < 0.05) to be the primary driver of microbial diversity which is consistent with several studies that report pH as the major predictor of microbial diversity in some hydrothermal systems (Oliverio et al., 2018; Power et al., 2018). Results also showed that Eh had a significant correlation (Shannon: *R*^2^ = 0.62; Nonpareil: *R*^2^ = 0.43, *p* < 0.05) with diversity. Temperature on the other hand had minimal effect on diversity (Shannon: *R*^2^ = 0.12, *p* = 0.09; Nonpareil: *R*^2^ = 0.02, *p* = 0.25). This is in contrast to previous studies that reported a strong relationship between temperature and microbial diversity in hot springs (Miller et al., 2009; Sharp et al., 2014). Comparably, minimal effect of temperature on alpha diversity has been reported for hot spring communities in Yellowstone, United States, Fludir, Iceland and Tibetan Plateau, China (Wang et al., 2013; Podar et al., 2020).

### Microbial community composition of Solfatara-Pisciarelli hydrothermal systems

**Figure 6:**
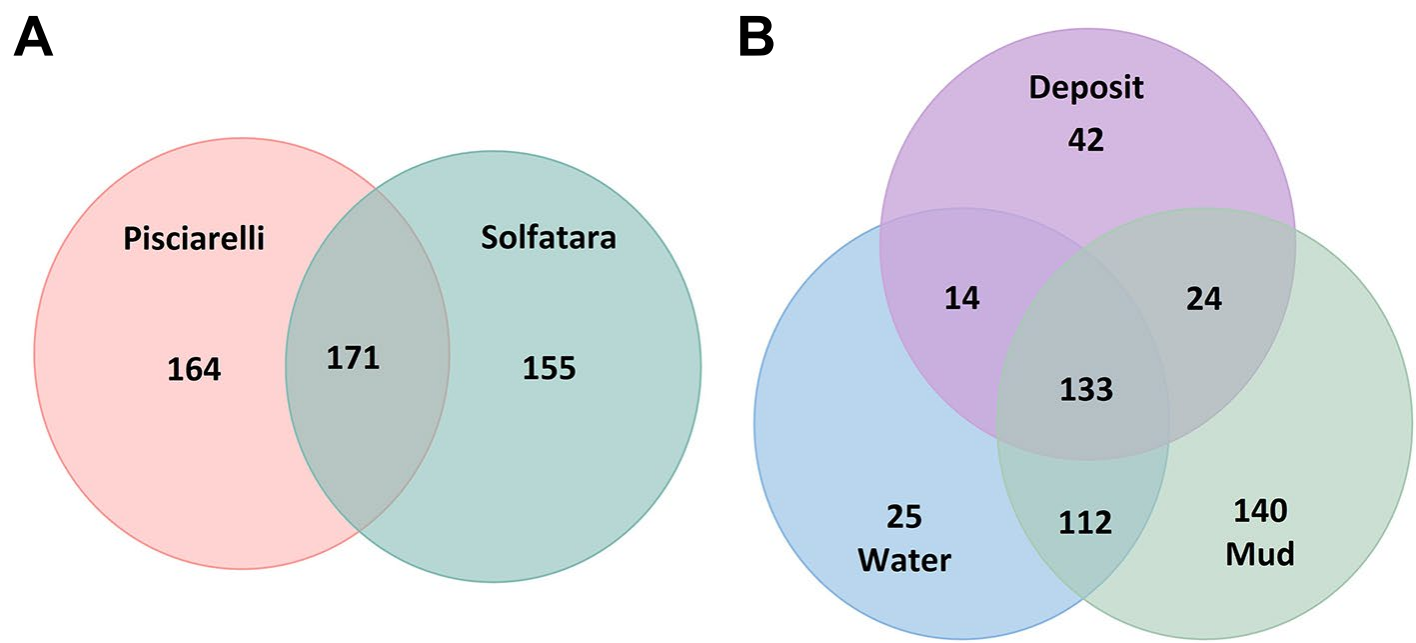
Venn diagram showing the number of unique and shared species between **(A)** two locations and **(B)** different types of substrates.

**Figure 7:**
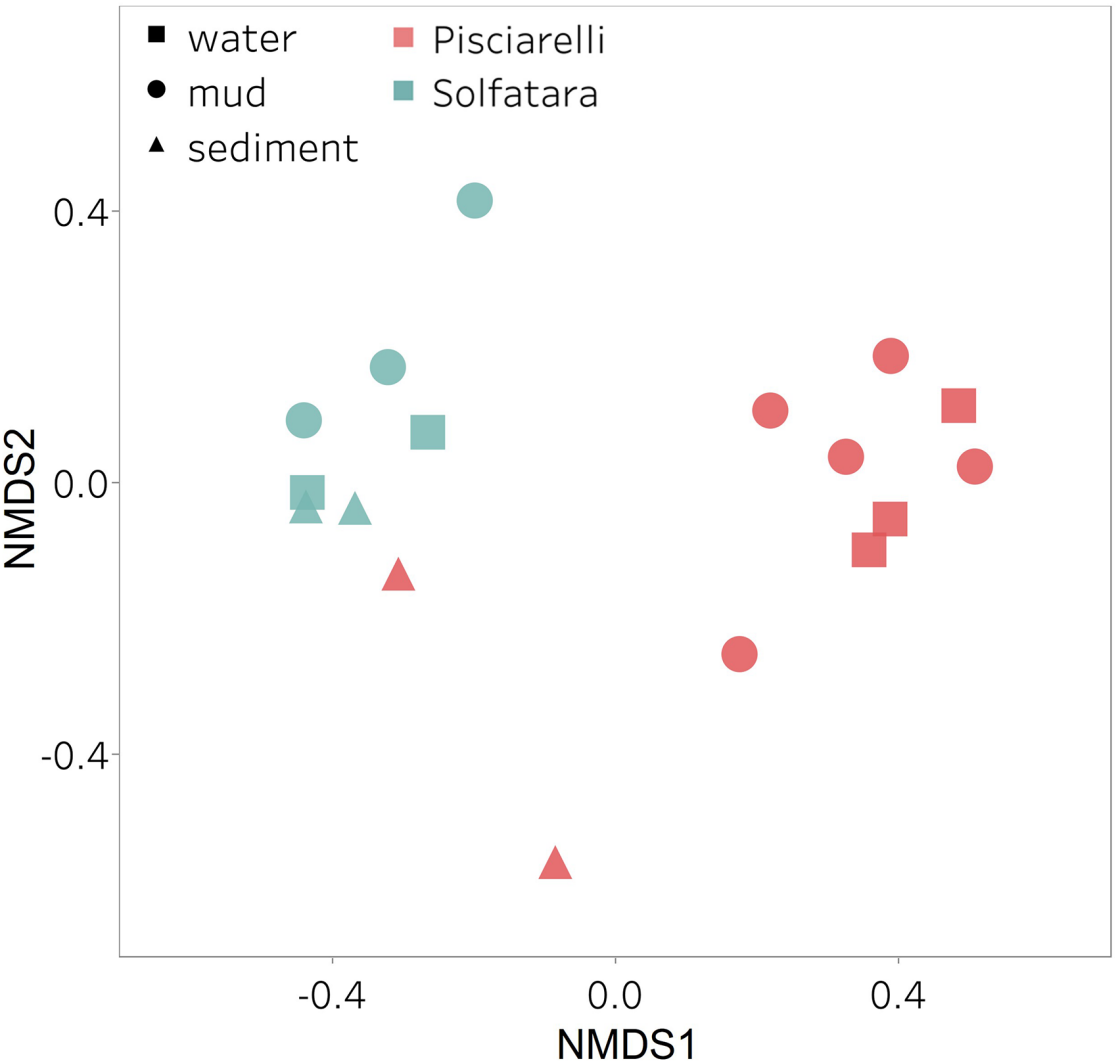
Nonmetric Dimensional Scaling (NMDS) ordination based on Bray–Curtis dissimilarity matrix. Bray–Curtis dissimilarity was calculated using species abundance from metagenomic profiling. The shapes define substrate types (water, mud, and deposits) and the colors represent locations (Pisciarelli and Solfatara).

After removing kitome and all potential contaminants, a total of 490 species (245 genera) were identified across all samples corresponding to 11 archaeal, 31 bacterial, 10 eukaryotic, and four viral phyla (Supplementary Table 6). Our results showed that overall, 33% (133) of the species detected were shared across the different types of substrates but each substrate contained unique species among which water had the least number of unique taxa and mud contained the highest (Figure 6B). NMDS plot (Figure 7) generated based on Bray–Curtis dissimilarity matrix revealed that the microbial community structure of Pisciarelli was distinct from Solfatara (ANOSIM: *R* = 0.63 for species; *R* = 0.67 for genus, *p* < 0.05). On average, the most abundant phylum at Pisciarelli was *Crenarchaeota* (46%) followed by *Proteobacteria* (5%) and *Actinobacteria* (2%), whereas Solfatara was dominated by *Firmicutes* (23%), *Proteobacteria* (21%), and *Actinobacteria* (12%; Figure 3B). In addition, Pisciarelli substrates had a higher number of viral sequences (4–49%) than Solfatara (1–4%; Supplementary Figure 2). Interestingly, we detected *Cyanobacteria* (0.5 to 4%) especially at Solfatara even in samples (SF-D_and SF-D_b) with a temperature (89°C) that exceeds the known temperature limit for photosynthesis in acidic environments (Brock, 1985; Cox et al., 2011; Hamilton et al., 2012). The *Cyanobacteria* we detected may have been introduced into the hydrothermal system from surrounding cooler environments (by wind or fluid circulations). However, we cannot decipher with certainty if they were environmentally introduced or if they are true members of the Solfatara-Pisciarelli hydrothermal community. We observed that at the species level, both locations shared 171 species (35%), however, there were over 100 species unique to each location (Figure 6A). Species unique to Pisciarelli include thermophiles such as *Pyrolobus fumarii*, *Pyrobaculum aerophilum* and *Metallosphaera sedula*. Unique species identified at Solfatara include *Thermaerobacter* sp. *FW80*, *Methylacidiphilum kamchatkense* and *Ilumatobacter coccineus*.

Our results showed that the microbial community structure in the high temperature environments (74–95°C) of Pisciarelli was different. The source of Pisciarelli large mud pool (84°C) was dominated by *Acidianus* (21–68%), *Pyrobaculum* (11–21%) and *Bicaudavirus* (11–43%) but as temperature decreased away from the source (79°C), we detected a decrease in the abundance of *Acidianus* (20–23%) and *Bicaudavirus* (15–17%) but an increase in the abundance of *Pyrobaculum* (30–43%) in the discharge channel. Water from Pisciarelli small mud pool (89°C) was also dominated by *Acidianus* (48–59%), but *Pyrobaculum* accounted for <1% of total sequences in this mud pool. Interestingly, the viral genus, *Ampullavirus* accounted for over 80% of sequences in Pisciarelli epilithic microbial layer (74°C) and for 26% of sequences in both Pisciarelli fumarolic deposit (93°C) and mud from Pisciarelli small mud pool (88°C). Most of the viruses detected in Pisciarelli fumarolic deposits, epilithic microbial layer and mud from the small mud pool were known archaeal viruses (e.g., *Ampullavirus*, *Bicaudavirus*, and *Betalipothrixvirus*) even though archaea accounted for <3% of the total sequences in these samples. This difference in relative abundance between archaeal viruses and their known host suggests that each archaeal species may host more than one virus type or the archaeal viruses present in Solfatara-Pisciarelli CHSs may have a broader host range that includes bacteria or/and eukaryotes (Munson-McGee et al., 2018). When substrates were compared at each location, we observed that Pisciarelli substrates harbored significantly different (ANOSIM species: *R* = 0.64; genus: *R* = 0.62, *p* < 0.05) microbial communities. SIMPER analysis showed that the sulfur-oxidizing archaeal genus *Acidianus* was significantly (*p* < 0.05) more abundant in Pisciarelli water communities, the archaeal genus *Pyrobaculum* showed a higher relative abundance in mud communities, and *Ampullavirus* showed a higher relative abundance in fumarole deposits communities (Supplementary Table 9; Figure 4B). Overall, Pisciarelli water and mud communities were similar but distinct from communities in Pisciarelli fumarolic deposits. The distinction between communities in the mud pools and fumarolic deposits at Pisciarelli may be linked to fluids and/or may reflect the availability of oxygen. Although the entire Pisciarelli environment has high temperatures and is highly reducing, the vigorous mixing of hydrothermal gas with air may create pockets of atmospheric O_2_ in pore spaces of fumarolic deposits which support aerobic communities that may be absent or in low abundance in mud pools. In contrast, high temperatures of mud pools inhibit the dissolution of oxygen thereby creating microaerobic environments that may favor thermophilic facultative and obligate anaerobes such as *Acidianus* and *Pyrobaculum*. Furthermore, the presence of *Pyrobaculum* specifically *Pyrobaculum arsenaticum* which is a strict anaerobe (Huber et al., 2000) and members of the genus *Sulfuracidifex* who are all obligate aerobes in Pisciarelli large pool may be an indication that conditions in this pool fluctuate between microaerobic and completely anaerobic, possibly in congruence with the intensity of hydrothermal activity.

At Solfatara, we observed variability in genus abundance across samples even between replicates (Figure 4B). For example, *Acidibacillus* accounted for 11% of sequences in SMP-M_a1 but was <1% in replicate mud sample, SMP-M_b. The difference between replicates may derive from the heterogeneous nature of Solfatara-Pisciarelli CHSs; however, seeing that genus abundance also varied in our sequencing replicates (SMP-M_a1 and SMP-M_a1.2), the variability in genus abundance may likely be bias introduced from sequencing. Results also showed that a large percentage of Solfatara sequences (20–36%) were unclassified at the genus level, suggesting a considerable amount of potentially novel genera at this environment. Interestingly, at Solfatara, we detected several genera that are not known thermophiles and not commonly found at pH < 2 (e.g., *Rhizopus*; 1.7–14%, *Actinomadura*; 1–9%, *Terasakiella*; 0.7–5.6%, and *Clostridioides*; 0.3–2.5%; Wang et al., 2014; Strazzulli et al., 2020). These genera were present in Solfatara water (43°C), mud (69°C), and fumarolic deposits (89°C). Furthermore, we detected these genera in most Pisciarelli samples but to a lesser degree. These microorganisms may not be native to the hydrothermal systems but likely introduced into the habitat from extensive human activity around the crater. In addition, the fluids in mud pools are a mixture of hydrothermal fluids and meteoric water which could deliver genetic material from non-native microorganisms to the hydrothermal systems. This possible introduction of non-native microorganisms into CHSs may be a potential problem for microbial ecology studies since the very nature of the CHSs makes it difficult to completely exclude all environmental contaminants. In contrast to Solfatara water and fumarolic deposits communities, Solfatara mud communities was dominated by *Sulfobacillus* (10–34%) and *Acidibacillus* (4–20%). However, unlike Pisciarelli, the differences across Solfatara substrates were not significant (ANOSIM species: *R* = 0.03, *p* = 0.50; genus: *R* = 0.03, *p* = 0.43) suggesting that variations in microbial community structure across substrates is not a general phenomenon but is specific to hydrothermal systems and possibly linked to the physical and geochemical conditions of individual systems (Wang et al., 2014). The microbial community structure observed in our study is in contrast with studies that investigated the microbial ecology of Solfatara-Pisciarelli CHSs and identified *Acidithiobacillus* and *Metallosphaera* as the dominant genus in Solfatara and Pisciarelli, respectively (Iacono et al., 2020; Strazzulli et al., 2020). Furthermore, the dominance of archaea which we observed in Pisciarelli mud pools is in contrast with a study that reported a low archaea/bacteria ratio in high temperature waters (>85°C) of Pisciarelli (Crognale et al., 2022). This contrast between our study and other Solfatara-Pisciarelli studies is likely the consequence of a microbial diversity that reflects differences in sampling year/periods from active CHSs with variable geochemistry. Additionally, the dissimilarity may be related to differences in sampling points (10 cm depth vs. 1 cm depth), sample handling, DNA extraction protocol, bias introduced by 16S rRNA gene primers or sequencing technology. An assessment of the effects of environmental variables on microbial community composition showed that pH and Eh (Mantel: *ρ* = 0.53 and *ρ* = 0.47, respectively, *p* < 0.05) had the strongest correlation to beta diversity which is consistent with reports from other studies that pH and redox potential shape microbial community composition (Alsop et al., 2014; Power et al., 2018). The concentrations of NH_4_^+^ and SO_4_^2−^ (Mantel: *ρ* = 0.38 and *ρ* = 0.23, respectively, *p* < 0.05) also had significant correlations to beta diversity. However, temperature had no significant (Mantel: *ρ* = 0.1, *p* = 0.14) effect on the microbial community composition of Solfatara-Pisciarelli CHSs, which coincides with temperature trends observed for alpha diversity.

### Diversity, abundance and metabolic potential of Solfatara-Pisciarelli MAGs

**Figure 8:**
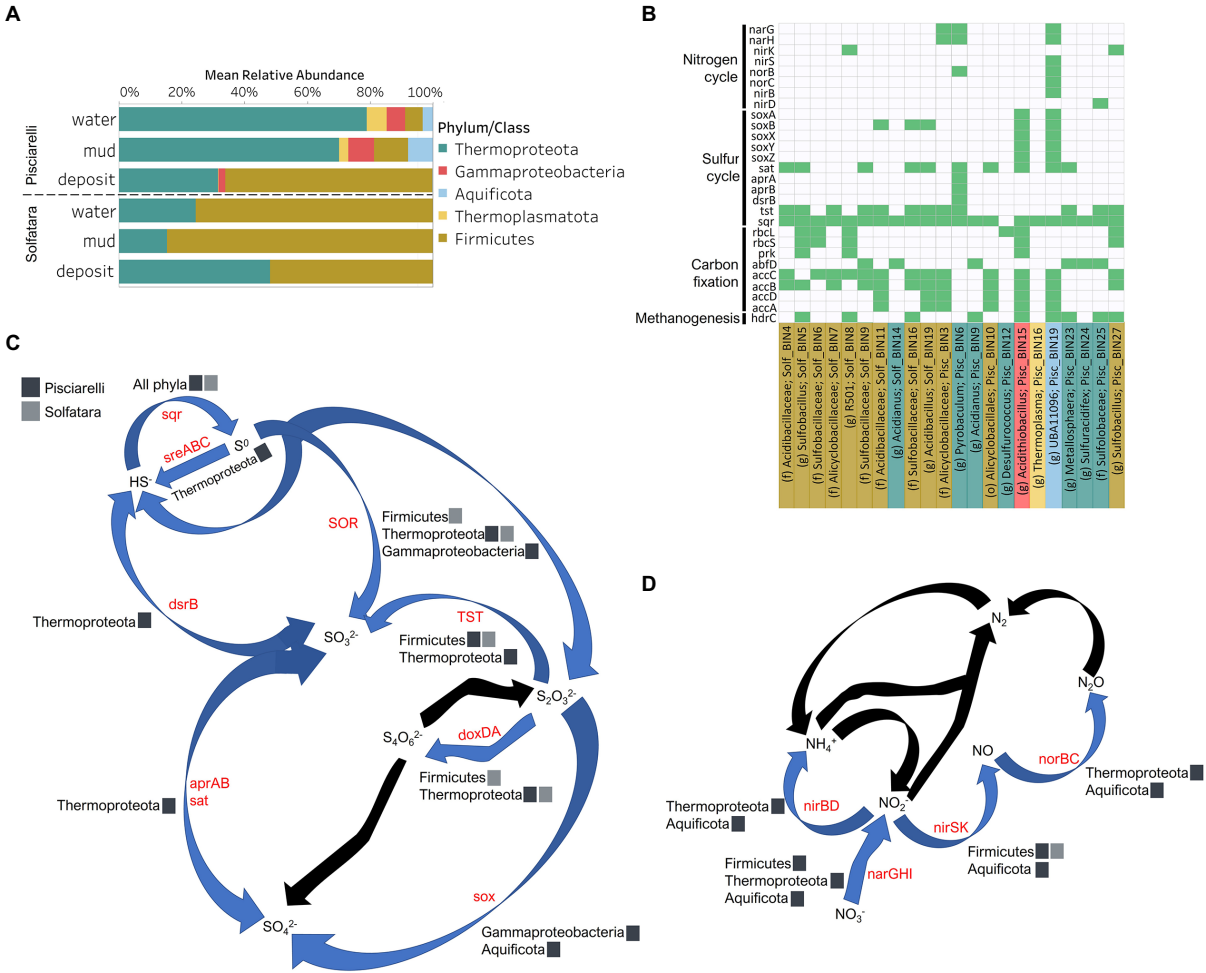
Taxonomy and functional potential of Metagenome Assembled Genomes (MAGs) in Solfatara-Pisciarelli hydrothermal systems. **(A)** Normalized mean (relative) abundance of sequences assigned to each MAG at the phylum/class level in each substrate. **(B)** Tile plot of selected marker genes (*y*-axis) present in each MAG (*x*-axis). Green color represents presence of gene in MAG. MAGs on the *x*-axis are colored based on phylum/class. Each MAG is labeled either by genus level assignment (g) or by the lowest taxonomic level assigned (f; family and o; order) to the MAG. **(C)** Sulfur cycle and **(D)** Nitrogen cycle. Phyla/Class from the different locations participating in each process are indicated. Gene participating in each process are indicated in red text. Black arrows denote processes that were not found in MAGs.

To identify the metabolic potential of members of the microbial community and their connection to geochemistry, we generated MAGs. After co-assembly and binning, a total of 22 MAGs (10 from Solfatara and 12 from Pisciarelli) with >50% completeness and <10% contamination were recovered. Out of the 22 MAGs recovered, 16 had completeness >90% and contamination <5%. Taxonomic classification revealed that MAGs belonged to three bacterial (*Firmicutes*, *Proteobacteria*, and *Aquificota*) and 2 archaeal (*Thermoproteota* also known as *Crenarchaeota* and *Thermoplasmatota*) phyla (Supplementary Tables 10, 11), with over 40% (9 out of 22) of unclassified genomes at the genus level suggesting that our MAGs represent novel genera. MAGs belonging to the phylum *Thermoproteota* were most represented in Pisciarelli water and mud while *Firmicutes* were more abundant in all Solfatara substrates and Pisciarelli fumarolic deposits (Figure 8A), which is consistent with our results from metagenome fragments. On average, the most abundant MAGs in Pisciarelli water and mud belong to species of the *Acidianus* and *Pyrobaculum* genera and an unknown species of the family *Sulfolobaceae* whereas the most abundant MAGs in fumarolic deposits belong to 2 unknown species: one species of the *Alicyclobacillaceae* family and the other species of the *Alicyclobacillales* order and a third species belonging to the *Acidianus* genus (Supplementary Table 11). In contrast, the most abundant MAG in all Solfatara samples belong to species of the *Acidianus* genus. When MAGs were dereplicated based on FastANI of ≥95% we found that only 2 species (*Acidianus infernus*; and an unknown species of the *Alicyclobacillaceae* family) were shared between locations further confirming that although an overlap exists between both locations; they have different microbial community structures. Since acidic hydrothermal environments are dominated by chemolithoautotrophic communities that derive energy from inorganic compounds, we analyzed MAGs for marker genes involved in carbon fixation, sulfur, nitrogen, and methane metabolism.

#### Carbon Metabolism

**Figure 9:**
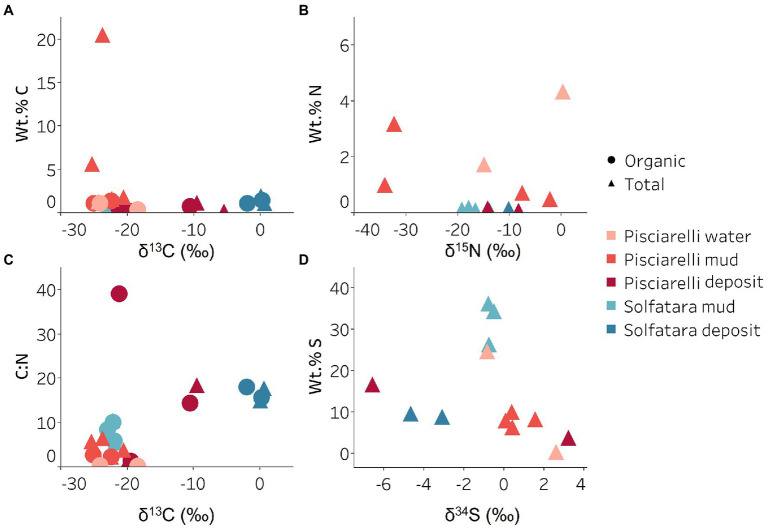
Carbon, nitrogen, and sulfur isotope values measured in Solfatara and Pisciarelli hydrothermal systems. **(A)** Total Carbon (TC) wt.%, Total Organic Carbon (TOC) wt.% and δ^13^C values. The wt.% values of TC and TOC are very similar indicating that carbon measured in the samples is mostly organic. The δ^13^C values exhibit a wide range of values. **(B)** Total Nitrogen wt.% and δ^15^N values. Pisciarelli mud and water have the highest concentrations of N. The δ^15^N values exhibit a large variation with values ranging from 0.41 to −34.04‰. **(C)** C:N ratio and δ^13^C values both point out that most of the carbon measured shows values typical for microorganisms with various carbon fixation pathways. **(D)** Total Sulfur wt.% and δ^34^S exhibit values characteristic for oxidized fumarolic H_2_S and sulfur with sedimentary origin.

Organic and inorganic carbon isotope (wt% and δ^13^C) values (Figures 9A,C) were very similar indicating that carbon measured at both locations is mostly organic and falls within the range typical for microorganisms with varied carbon fixation pathways (Sharp, 2007; Havig et al., 2011), which is also indicated by the functional potential of our MAGs. Genes encoding the key enzymes for Calvin-Benson-Bassham (CBB) cycle, ribulose-biphosphate carboxylase (*rbc*) and phosphoribulokinase (*prk*) were found in *Firmicutes* MAGs (*Sulfobacillus* and *R501*) as well as *Gammaproteobacteria* MAG (*Acidithiobacillus*; Supplementary Table 12; Figure 8B) similar to what has been reported in other studies (Caldwell et al., 2007; Cerqueira et al., 2018). Owing to the abundance of *Firmicutes* in Pisciarelli fumarolic deposits and Solfatara microbial communities, CBB may be the primary mode of carbon fixation in these environments. The *rbc* gene was also detected in an archaeal MAG (*Desulfurococcus*); however, in archaea, this gene encodes enzymes reported to be involved in the reductive hexulose-phosphate pathway (Kono et al., 2017). The marker gene encoding 4-hydroxybutyryl-CoA dehydratase (*abfD*), an enzyme in 3-hydroxypropionate/4-hydroxybutyrate (3-HP/4-HB) cycle, was present in all MAGs of the *Sulfolobaceae* family (*Acidianus*, *Metallosphaera*, *Sulfuracidifex*) which is consistent with the presence of the 3-HP/4-HB cycle in the crenarchaeal order *Sulfolobales* (Berg et al., 2010). *Crenarchaeota* especially the order *Sulfolobales* were abundant in Pisciarelli water and mud; hence, 3-HP/4-HB cycle may be the primary mode of carbon fixation in Pisciarelli mud pools. The *abfD* gene was also detected in one *Firmicutes* MAG (unknown species of the *Sulfobacillaceae* family) from Solfatara. The 3-HP/4-HB cycle to the best of our knowledge has not been reported in *Sulfobacillaceae* and the absence of other genes encoding enzymes involved in the 3-HP/4-HB cycle implies that this cycle may be absent in this *Sulfobacillaceae* MAG. One possible explanation is that the enzyme encoded by the *abfD* gene in this *Sulfobacillaceae* MAG may be involved in an alternative metabolic pathway like has been reported for *Clostridium* (Scherf and Buckel, 1993; Scherf et al., 1994). Another possible explanation is that the *Sulfobacillaceae* MAG may have acquired the *abfD* gene *via* horizontal gene transfer.

Genes encoding the key enzymes in Wood-Ljungdahl (WL) pathway and 3-hydroxyprioponate (3-HP) bicycle were absent in all MAGs and in the entire metagenome dataset, suggesting that both pathways may not be important for carbon fixation at both locations. The marker gene encoding ATP citrate lyase (*aclAB*), an enzyme involved in reductive tricarboxylic acid (rTCA) cycle, was also absent in all MAGs. ATP citrate lyase catalyzes citrate cleavage in rTCA cycle but in *Aquificaceae*, this reaction is catalyzed by the combined action of citryl-CoA synthetase (*ccsAB*) and citryl-CoA lyase (*ccl*) (Aoshima et al., 2004a,b) which were found in our *Aquificota* MAG (*UBA11096*) from Pisciarelli metagenome indicating potential for rTCA.

Potential for methanogenesis was absent in MAGs from both locations. Although the gene encoding hydrogen disulfide reductase (*hdrABC*) involved in methanogenesis was found in *Firmicutes* (*Sulfobacillus*, *R501*, and unknown species of the *Sulfobacillaceae* family), *Gammaproteobacteria* (*Acidithiobacillus*), *Aquificota* (*UBA11096*), and *Thermoproteota* (*Metallosphaera*) MAGs, the gene encoding key enzyme, methyl-CoM reductase (*mcr*) in methanogenesis was absent. Given the absence of *mcr* gene, the absence of genes encoding other enzymes involved in methanogenesis especially in the *Metallosphaera* MAG that was 100% complete, and the absence of δ^13^C values lower than −30‰, it is likely that the *hdrABC genes* in these MAGS are not used in methanogenesis but are most likely used in sulfur metabolism (Mander et al., 2004; Wang et al., 2019). Potential for methane oxidation (methanol dehydrogenase; *mdh1* and particulate methane monooxygenase; *pmo*) was also absent in all MAGs.

#### Sulfur metabolism

Sulfur isotope values (δ^34^S) measured in deposits from both locations (Figure 9D) are characteristic of sulfur with sedimentary origin, but δ^34^S values (−0.83 to 2.62‰) measured in water and mud where oxidation of H_2_S is extensive, fall within the range for SO_4_^2−^ formed from sulfide oxidation (−2.5 to 2.4‰; Allard et al., 1991). In acidic environments like Solfatara and Pisciarelli, abiotic oxidation of H_2_S-rich gas produced from the disproportionation of volcanic SO_2_ is inhibited (D’Imperio et al., 2008), thus providing a great habitat for sulfur-oxidizing microorganisms that utilize H_2_S for energy generation. The abundance in H_2_S is consistent with the presence of genes encoding enzymes involved in the oxidation of a variety of reduced sulfur compounds (H_2_S, S^0^ or S_2_O_3_^2−^) at both locations. Genes encoding the enzyme, sulfide:quinone oxidoreductases (*sqr*) for H_2_S oxidation to elemental sulfur (S^0^) were detected in all MAGs except one *Thermoproteota* MAG (*Desulfurococcus*; Figures 8B,C). The presence of *sqr* gene in most MAGs is indicative of the importance of H_2_S oxidation to microbial communities at both locations. S^0^ formed can be reduced to H_2_S by the enzyme molybdopterin sulfur reductase (*sreABC*) or disproportionated to H_2_S, thiosulfate (S_2_O_3_^2−^) and sulfite (SO_3_^2−^) by sulfur oxygenase reductase (*SOR*). The *sreABC* gene was found in *Thermoproteota* MAG (*Acidianus*) while *SOR* gene was found in *Firmicutes* (*Sulfobacillus*, *R501*, and *Acidibacillus*) *Gammaproteobacteria* (*Acidithiobacillus*) and *Thermoproteota* (*Acidianus* and *Sulfuracidifex*) MAGs.

Thiosulfate (S_2_O_3_^2−^) may not be available for microbial metabolism since it disproportionates to S^0^ and SO_3_^2−^ at pH < 4 (Nordstrom et al., 2004); however, microbial communities exhibited potential to utilize S_2_O_3_^2−^. Thiosulfate can be oxidized to SO_4_^2−^ *via* the *Sox* system (*soxXYZABCD*) which we detected in *Gammaproteobacteria* (*Acidithiobacillus*) and *Aquificota* (*UBA11096*) MAGs. In both MAGs, *soxCD* was absent suggesting that they oxidize S_2_O_3_^2−^ to S^0^ instead of SO_4_^2−^ which is consistent with studies that identified the *sox* cluster without *soxCD* in *Acidithiobacillus* (Wang et al., 2019). Thiosulfate can also be oxidized to tetrathionate (S_4_O_6_^2−^) by thiosulfate:quinol oxidoreductase (*doxDA*) or reduced to SO_3_^2−^ by thiosulfate:cyanide sulfur transferase (*tst*). The *doxDA* gene was found in all *Sulfolobaceae* MAGs (*Acidianus*, *Metallosphaera*, *Sulfuracidifex*) and in the *Firmicutes* (*R501*) MAG, while *tst* gene was found in 2 *Thermoproteota* (*Pyrobaculum*, *Metallosphaera*) MAGs and most *Firmicutes* MAGs. H_2_S can be regenerated by reducing SO_4_^2−^ *via* dissimilatory sulfate reduction (DSR). Genes encoding enzymes (dissimilatory sulfite reductase; *dsrAB*, adenylylsulfate reductase; *aprAB*, and sulfate adenylyltransferase; *sat*) involved in DSR were detected in one *Thermoproteota* (*Pyrobaculum*) MAG. The presence of both genes encoding enzymes involved in sulfur oxidation and reduction in some MAGs implies that microbial communities in Solfatara-Pisciarelli systems may take advantage of the high energy yield that results from coupling sulfur oxidation to sulfur reduction, which has been previously observed in other microbial community studies (Glamoclija et al., 2004; Crognale et al., 2022). Our results are consistent with studies that found the abundance of organisms capable of utilizing sulfur (Inskeep et al., 2013) and an enrichment of genes (*sqr*, *doxDA*, and *SOR*) involved in metabolizing reduced sulfur compounds in acidic hot springs (Colman et al., 2019a).

#### Nitrogen metabolism

Our results showed that nitrogen metabolism may not be an important energy-yielding pathway for microbial communities even in Pisciarelli mud pools where NH_4_^+^ was abundant (Figures 8B,D). Exceptionally low δ^15^N values were observed at both locations (Figure 9B) and the lowest δ^15^N values ever measured were found in the Pisciarelli epilithic microbial layer. These extremely low δ^15^N values are difficult to explain in the context of metabolic potential present in our MAGs or metagenome fragments. Low and negative δ^15^N values are sometimes linked to microbial ammonia oxidation and/or nitrogen fixation (Tozer et al., 2005; Havig et al., 2011). Although Crognale et al. (2022) reported ammonia oxidation to be the most likely metabolic pathway in Pisciarelli mud pools due to its high energy yield, the absence of genes encoding enzymes involved in ammonia oxidation (ammonia monooxygenase; *amoCAB* and hydroxylamine dehydrogenase; *hao*) or nitrogen fixation (*nifD*, *nifK*, and *nifH*) in our MAGs and metagenome dataset indicates that microbial nitrogen metabolism cannot explain the geochemistry we observed. Previously observed negative δ^15^N values (about −10‰) have been associated with the dissolution of magmatic nitrogen in water at elevated temperatures and pressures (Labidi et al., 2020). While it is very likely, the mechanism by which magmatic nitrogen, high temperature and pressure would contribute to the extremely low δ^15^N values measured at Solfatara and Pisciarelli is unclear.

Evidence of denitrification which has not been linked to low and negative δ^15^N values was found in five MAGs (*Sulfobacillus*, *R501*, unknown species of the *Alicyclobacillaceae* family, *Pyrobaculum*, and *UBA11096*). However, none of the MAGs carried all the genes encoding enzymes for the complete denitrification pathway (nitrate reductase, *narGH* or *napA*; nitrite reductase, *nirK*; nitric oxide reductase, *norB*; nitrous oxide reductase, *nosZ*). The complete set of genes for denitrification were also absent in metagenome fragments. One Pisciarelli MAG (*UBA11096*) belonging to the family *Aquificaceae*, carried most of the denitrification genes except *nosZ* which is consistent with studies that found nitrous oxide (N_2_O) to be the end-product of denitrification in members of *Aquificaceae* (Nakagawa et al., 2004). Dissimilatory nitrate reduction to ammonia *via* nitrite reductases (*nirBD*) was detected only in the *Aquificaceae* MAG from Pisciarelli.

## Conclusion

Results from this study showed that Solfatara and Pisciarelli are lithologically similar but geochemically distinct. At Pisciarelli, we observed varying geochemistry among mud pools and fumarolic deposits, which results from subsurface fluid–rock interactions, whereas Solfatara substrates were geochemically similar. Shannon diversity was significantly different between locations but showed no significant difference across substrates. We found pH to be the most important driver of alpha diversity and microbial community composition. NMDS plot and ANOSIM showed that Solfatara’s microbial community structure exhibited no significant difference across substrates which also coincides with observed trends in geochemistry. In contrast, at Pisciarelli, microbial community structure followed trends in fluid availability rather than geochemical trends. Geochemically, water and mud from Pisciarelli large mud pool and water from Pisciarelli small mud pool clustered distinctly from fumarolic deposits, discharge channel mud and mud from Pisciarelli small pool. However, based on microbial communities, all water and mud samples clustered distinctly from fumarolic deposits that were the driest substrates analyzed; results showed that Pisciarelli fumarolic deposits were more similar to Solfatara samples than samples from Pisciarelli mud pools. Overall, the genus *Acidianus* dominated Pisciarelli water and *Pyrobaculum* was more abundant in Pisciarelli mud. Interestingly, Pisciarelli fumarolic deposits, epilithic microbial layer, and mud from small mud pool had a high number of viral sequences. Solfatara and Pisciarelli had distinct microbial communities; on average, *Acidianus* and *Pyrobaculum* dominated Pisciarelli while *Sulfobacillus* and *Acidibacillus* were dominant at Solfatara. Although microbial communities were distinct between the two locations and between Pisciarelli substrates, MAGs indicated functional redundancy. For example, the potential to reduce H_2_S to S^0^ was observed in almost all MAGs. Further, MAGs showed that microbial communities inhabiting both locations took advantage of the major volcanic gases (CO_2_ and H_2_S) *via* carbon fixation and sulfur oxidation. The 3HP/4-HB pathway found in most archaeal MAGs is most likely the primary mode of carbon fixation in Pisciarelli mud pools dominated by (hyper)thermophilic archaea whereas CBB cycle is most likely the primary carbon fixation pathway in all other environments where bacteria was abundant. At both locations, sulfur cycling is represented by oxidation of H_2_S-rich volcanic gas and other reduced sulfur compounds. Comparative geochemical and metagenomic analyses demonstrate that ecological differences across substrates are not a widespread phenomenon but specific to the system. Therefore, this study demonstrates the importance of analyzing different substrates of CHSs to understand the full range of microbial ecology to avoid biased ecological assessment.

## Data availability statement

The datasets presented in this study can be found in online repositories. The names of the repository/repositories and accession number(s) can be found below: https://www.ncbi.nlm.nih.gov/, Bioproject accession PRJNA889931.

## Author contributions

MG, AS, MF, GN, and CT designed and organized the project. MG, RS, MP, AM collected the samples. IU, RB, MF, and MG collected the data. IU, MG, and MF contributed to the data processing and analyses. IU and MG wrote the manuscript. All authors contributed to the article and approved the submitted version.

## Funding

This research was enabled through the Alfred P. Sloan Foundation’s support of the Deep Carbon Observatory Deep Earth Carbon Degassing program (DECADE) to MG and Rutgers Faculty Program Start Up. IU was supported by Rutgers University Transform Graduate Fellowship.

## Conflict of interest

The authors declare that the research was conducted in the absence of any commercial or financial relationships that could be construed as a potential conflict of interest.

## Publisher’s note

All claims expressed in this article are solely those of the authors and do not necessarily represent those of their affiliated organizations, or those of the publisher, the editors and the reviewers. Any product that may be evaluated in this article, or claim that may be made by its manufacturer, is not guaranteed or endorsed by the publisher.

## Footnotes

^1^ https://www.bioinformatics.babraham.ac.uk/projects/fastqc/

## Supplementary material

SUPPLEMENTARY FIGURE 1 Ballot based on Principal Component Analysis (PCA) using bulk elemental composition of mud and fumarolic deposits.

SUPPLEMENTARY FIGURE 2 Taxonomic profile of Solfatara and Pisciarelli microorganisms based on metagenomic profiling. Sequences not classified at the domain level and species with relative abundance less than 0.01% were grouped into unclassified and low abundance species. All kitome were grouped into contaminant. Samples collected from Pisciarelli large pool are denoted by PLP, Pisciarelli small pool by PSP, Pisciarelli fumarole by PF, Solfatara mud pool by SMP, and Solfatara fumarole by SF. W represents water, M for mud, MO for mud outlet, E for epilithic microbial layer from dry mud wall, and D for deposits samples. Samples labeled a and b are replicates sampled from the same spot. Sample SMP-M_a1 and SMP-M_a1.2 are sequencing replicates.

SUPPLEMENTARY FIGURE 3 Relative abundance of kitome families. Kitome families with average abundance of <1% were grouped into Others <1%.

SUPPLEMENTARY FIGURE 4 Estimation of the coverage of microbial communities in **(A)** Solfatara and **(B)** Pisciarelli. The empty circles on the curves represent community coverage estimate at the sequencing effort applied. The top and bottom horizontal red dashed indicate 100 and 95% average community coverage, respectively. The arrows are the Nonpareil diversity estimates.
