# Supplementary figures and images for: Comparative metagenomics at Solfatara and Pisciarelli hydrothermal systems in Italy reveal that ecological differences across substrates are not ubiquitous
Source: Front Microbiol. 2023 Feb 1;14:1066406. doi: 10.3389/fmicb.2023.1066406 (PMC9930910; doi:10.3389/fmicb.2023.1066406)

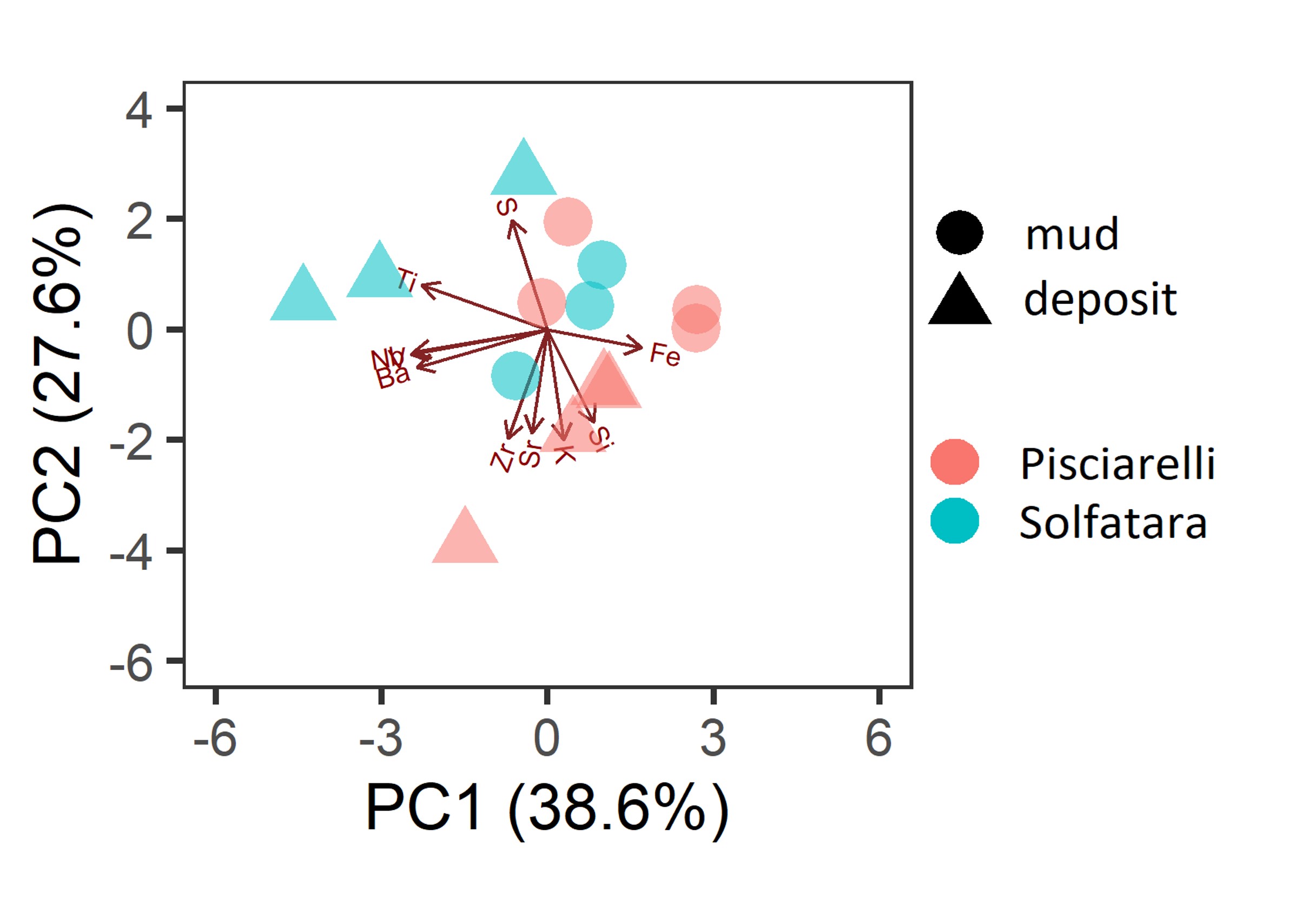

Supplement: SUPPLEMENTARY FIGURE 1 — Ballot based on Principal Component Analysis (PCA) using bulk elemental composition of mud and fumarolic deposits. [file Image_1.jpeg]

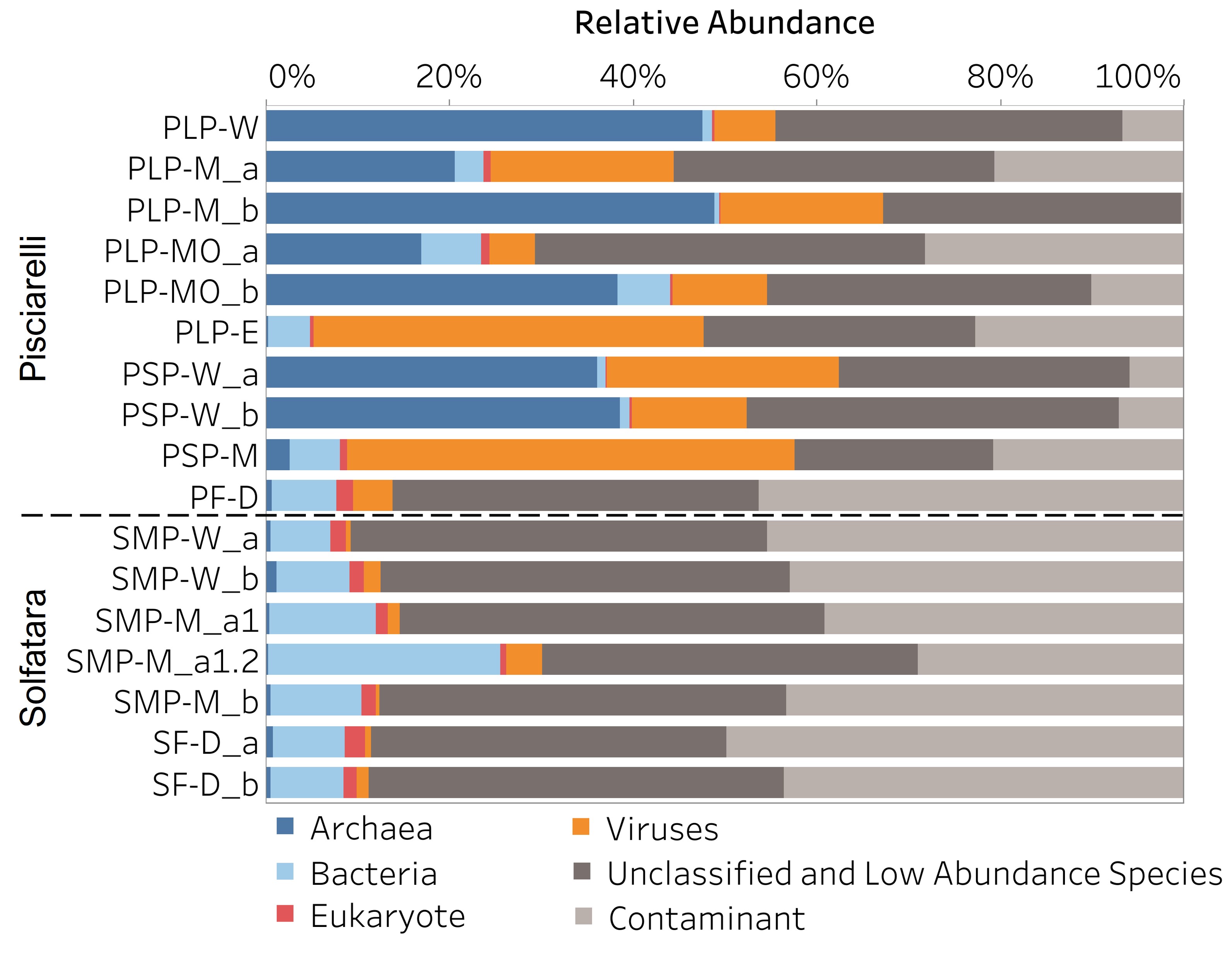

Supplement: SUPPLEMENTARY FIGURE 2 — Taxonomic profile of Solfatara and Pisciarelli microorganisms based on metagenomic profiling. Sequences not classified at the domain level and species with relative abundance less than 0.01% were grouped into unclassified and low abundance species. All kitome were grouped into contaminant. Samples collected from Pisciarelli large pool are denoted by PLP, Pisciarelli small pool by PSP, Pisciarelli fumarole by PF, Solfatara mud pool by SMP, and Solfatara fumarole by SF. W represents water, M for mud, MO for mud outlet, E for epilithic microbial layer from dry mud wall, and D for deposits samples. Samples labeled a and b are replicates sampled from the same spot. Sample SMP-M_a1 and SMP-M_a1.2 are sequencing replicates. [file Image_2.jpeg]

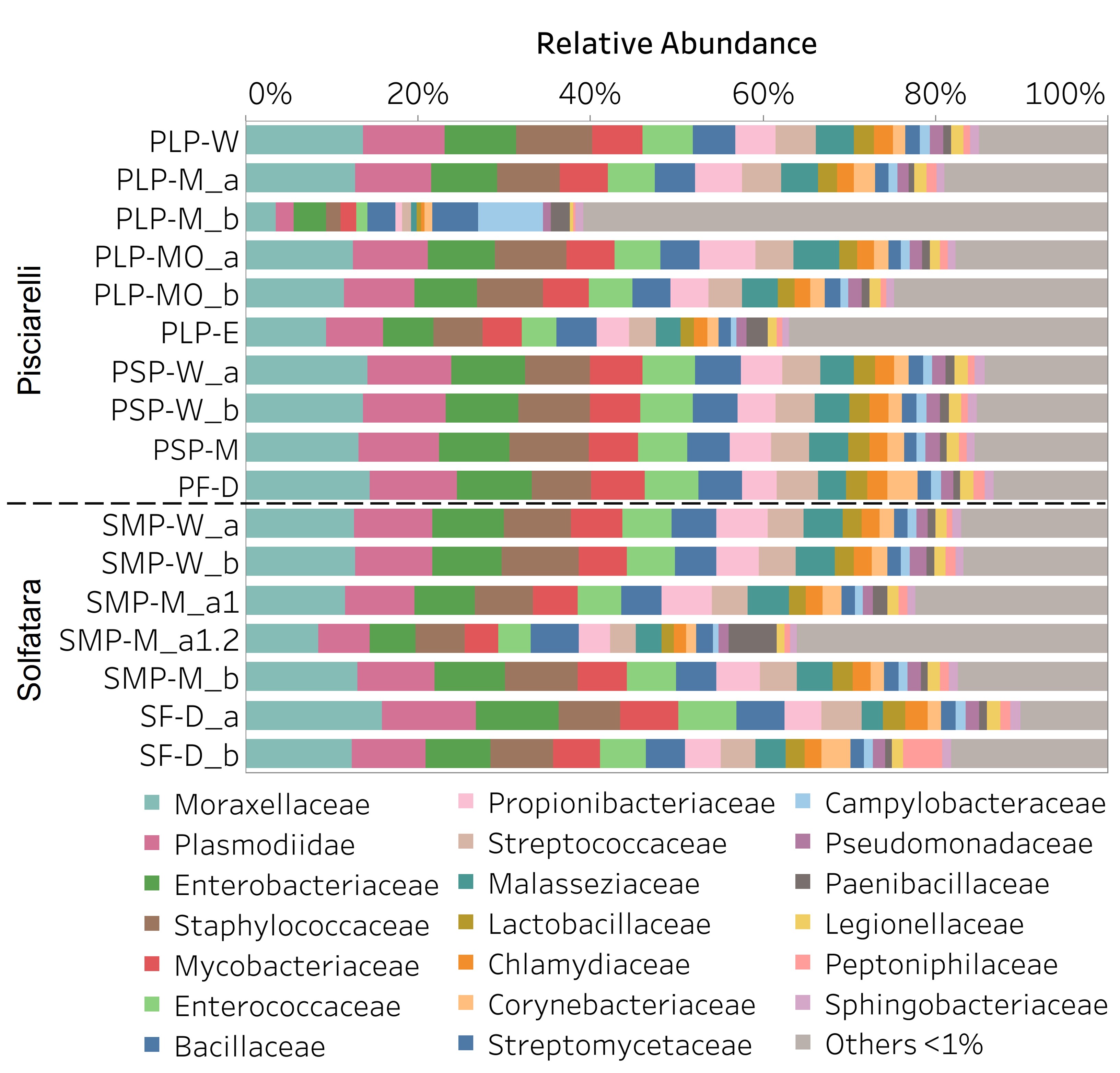

Supplement: SUPPLEMENTARY FIGURE 3 — Relative abundance of kitome families. Kitome families with average abundance of <1% were grouped into Others <1%. [file Image_3.jpeg]

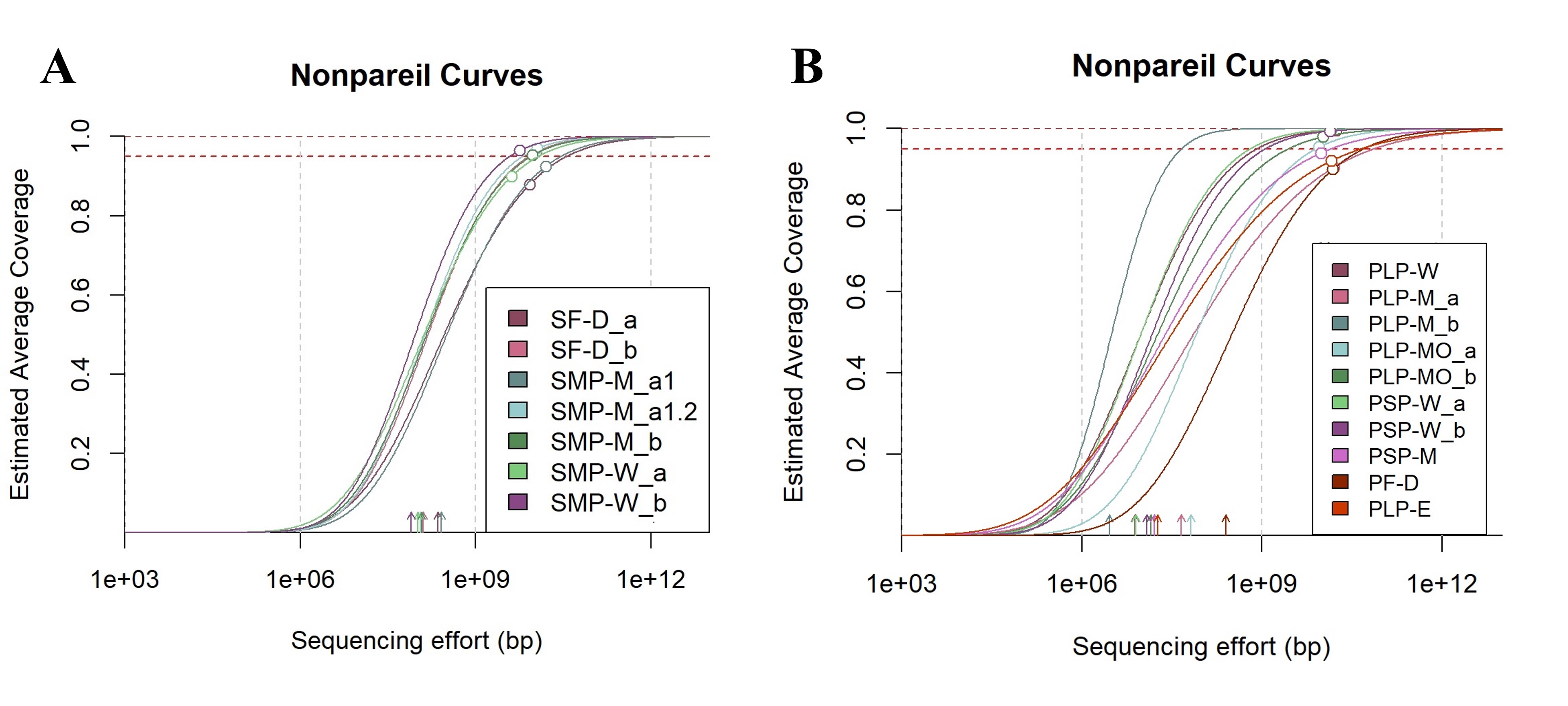

Supplement: SUPPLEMENTARY FIGURE 4 — Estimation of the coverage of microbial communities in (A) Solfatara and (B) Pisciarelli. The empty circles on the curves represent community coverage estimate at the sequencing effort applied. The top and bottom horizontal red dashed indicate 100 and 95% average community coverage, respectively. The arrows are the Nonpareil diversity estimates. [file Image_4.jpeg]
